# A Back-to-Back Gap Waveguide-Based Packaging Structure for E-Band Radio Frequency Front-End

**DOI:** 10.3390/mi16060644

**Published:** 2025-05-28

**Authors:** Tao Xiu, Zhi Li, Lei Wang, Peng Lin

**Affiliations:** 1 The 13th Research Institute of China Electronics Technology Group Corporation, Shijiazhuang 050057, China; xiut@bupt.cn (T.X.); lp9879@126.com (P.L.); 2 EMC Research Center, China Electronics Standardization Institute, Beijing 100007, China; 3 Faculty of Integrated Circuit, Xidian University, Xi’an 710071, China; lwang2020@stu.xidian.edu.cn

**Keywords:** bandpass filter, E-band, gap waveguide, power combiner

## Abstract

This paper presents our research on an E-band Radio Frequency (RF) front-end packaging structure based on back-to-back gap waveguide (GW). This design effectively mitigates the impact of air gaps on performance and offers the advantage of large assembly tolerances. Additionally, its back-to-back structure enables structural stacking, which can reduce the overall packaging size. In terms of functionality, the structure integrates hybrid couplers, bandpass filters, and amplifier packaging structures. Notably, the hybrid couplers provide high port isolation, facilitating a higher isolation duplex function by simply connecting high-order bandpass filters at the output ports without the need for additional optimization. Furthermore, these couplers also serve as power dividers/combiners. When combined with the H-plane amplifier packaging structures, the output power of the module is theoretically increased by 3 dB. Based on the measurements, the results indicate that this structure operates within the frequency ranges of 71–76 GHz and 81–86 GHz. The common port return loss is below 12 dB, while the in-band insertion loss is less than 2.26 dB and 2.42 dB, respectively. These findings demonstrate excellent electrical performance and suitability for E-band communication systems.

## 1. Introduction

With the rapid development of communication technology, the conventional microwave band has failed to meet the escalating demand for high-speed transmission. In recent years, the E-band (60–90 GHz) has emerged as a prominent frequency range usable for the enabling of high-speed wireless communication systems due to its abundant bandwidth resources compared to the microwave band and relatively lower atmospheric loss in comparison with the terahertz band. This frequency range finds extensive applications in 5 G fronthaul and backhaul links, as well as Space-Earth and Earth-Space communication links [1,2]. Moreover, owing to advancements in processing and packaging technology, E-band communication products are progressively being integrated and commercialized. Currently, low-power components such as frequency multipliers, mixers, and couplers can be seamlessly integrated into a single system-on-a-chip (SoC). However, integrating high-power components like power amplifiers and diplexers into chips re-mains challenging; hence, it becomes crucial to package diplexers and amplifiers within a module.

**Figure 1:**
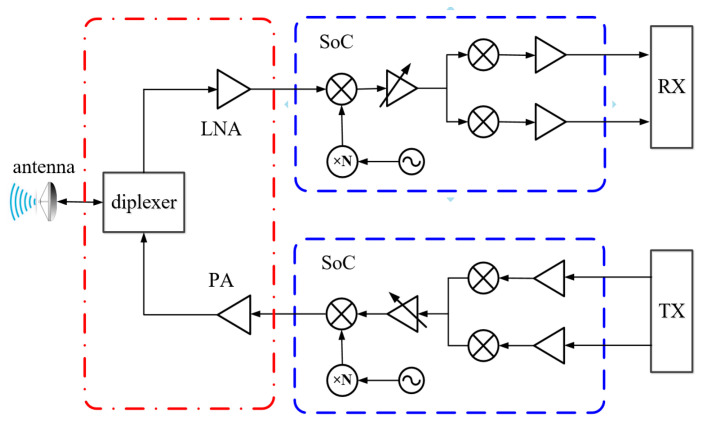
Topology of a typical FDD transceiver.

A typical E-band frequency division duplex (FDD) communication transceiver topology is shown in Figure 1; it consists of a diplexer, a low-noise amplifier (LNA), a power amplifier (PA), and two modulation links. Generally, in the E-band system, the modulation link takes the form of a chip. Therefore, this paper primarily focuses on integrating the LNA, PA, and diplexer components (as indicated by the red dash–dot circle in Figure 1). In this context, particular emphasis is placed on optimizing both the diplexer structure and the packaging configuration.

**Figure 2:**
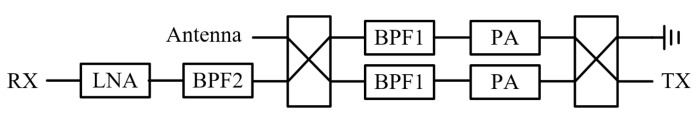
Schematic diagram of the constituent units of the proposed structure.

The diplexer plays an indispensable role in separating “receive” (RX) and “transmit” (TX) signals in the communication system. Typically, it consists of two bandpass filters (BPFs) and a power distribution unit, such as T-junctions [3,4], a manifold [5,6,7], hybrid couplers [8,9], circulator couplers [10], and so on. Among them, the T-junction and manifold structure are relatively simple and compact, resulting in lower insertion losses as well as improved amplitudes and group delays. However, the interactions between multiple branches’ BPFs can lead to design complexity and challenges. On the other hand, although hybrid-coupler diplexers and circulator-coupler diplexers have larger volumes, they offer a high level of isolation between each channel’s BPF, without any interaction, simplifying the design process [11]. Moreover, due to the power division effect of the hybrid coupler, the input power can be equally divided, which enables an increase in saturated output power for the module. As depicted in Figure 2, two PA chips can be separately packaged between the hybrid coupler and two BPFs to achieve power combination.

In recent years, there have been numerous advancements in chip and system pack-aging technology. Due to its advantages of low insertion loss and high-power capacity, the waveguide probe has always been the mainstream method of RF chip or system pack-aging. It can be categorized into H-plane probe structures [12,13,14] and E-plane probe structures [15,16]. Additionally, the gap waveguide (GW) structure has gained significant attention in packaging design owing to its ability to accommodate a certain air gap between the periodic metal structure and the top metal plate, for components not requiring complete electrical contact [17,18]. Components utilizing the gap waveguide structure have also been the subject of extensive research [19,20,21]. This research effectively addresses issues arising from air gaps and generated during the packaging process. Although the E-plane probe has the advantages of easy design and low insertion loss, its input/output ports are perpendicular to each other, which means that additional waveguide bending or other conversion structures are required, so the E-plane probe is not suitable for packaging, given the structure of the GW.

In this paper, our aim is to design a packaging structure for the RF front-end of a communication system that combines the advantages of back-to-back GW packaging and the power combination functions of the hybrid-coupled diplexer, while simultaneously providing frequency-selection and power combination functionalities. Its duplex function enables the FDD within the system, thereby enhancing the bidirectional transmission rate. Meanwhile, the power synthesis function increases the system’s output power, extending its communication range. Based on the measurements, the results show that the passbands are 70.95–76.2 GHz and 80.92–86.25 GHz, the common port return loss is below 12 dB, and the in-band insertion loss values are less than 2.26 dB and 2.42 dB, respectively.

## 2. Unit Design of the Packaging Structure

**Figure 3:**
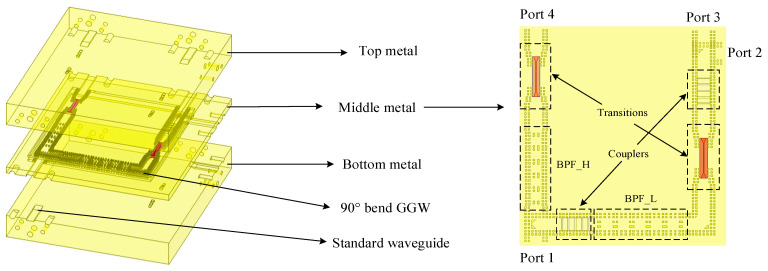
Processing model of the final packaging structure and diagram of the middle metal plate.

As shown in Figure 2 and Figure 3, the proposed packaging structure consists of a hybrid coupler with back-to-back GW structure, a 71–76 GHz and 81–86 GHz filter based on the GW structure, and a microstrip (MS)–GW transition. Since there is no interaction among the channels of the hybrid-coupled duplexer, we can design the required components separately and connect them directly, which greatly simplifies the design process.

### 2.1. Back-to-Back GW Design

**Figure 4:**
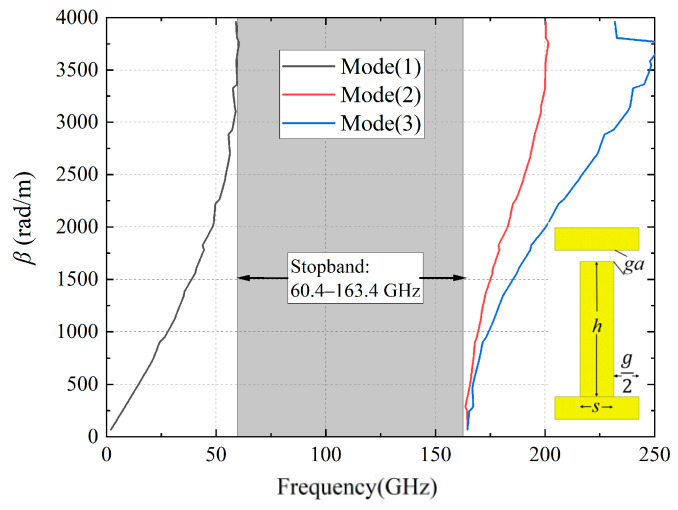
Dispersion diagram of the EBG.

The GW structure is composed of parallel metal plates and periodic nails. When the air gap between nails and plate is less than a quarter wavelength, it can be equivalent to an artificial magnetic conductor (AMC), effectively preventing electromagnetic wave leakage. As illustrated in Figure 4, with specific dimensions of *s* = 0.4 mm, *g* = 0.4 mm, *ga* = 0.05 mm, and *h* = 0.8 mm, the stopband ranging from 60.4 to 163.4 GHz is encompassed.

**Figure 5:**
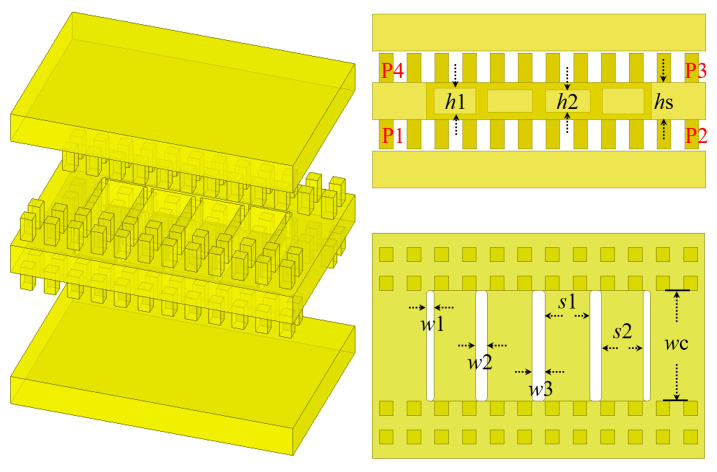
Structure and dimensions of the hybrid coupler, P1 to P4 represent four ports. (where *h*1 = 0.9 mm, *h*2 = 0.84 mm, *h*s = 1 mm, *w*1 = 0.22 mm, *w*2 = 0.34 mm, *w*3 = 0.38 mm, *s*1 = 1.29 mm, *s*2 = 1.2 mm, *w*c = 3.1 mm, and the radius of the chamfer is 0.1 mm).

As illustrated in Figure 5, the proposed back-to-back gap waveguide (GW) differs from other components of the multi-layer GW structure. It primarily consists of a central metal plate flanked by two layers of periodic nails. Covering metal plates are then added to both sides of this central plate, resulting in a back-to-back GW structure. This structure differs from the straightforward stacking of multi-layer gap waveguides. This design minimizes the effects of air gaps and assembly misalignment on performance, while also simplifying processing and assembly.

### 2.2. Hybrid Coupler and Bandpass Filter Design

The hybrid coupler plays a critical role in the design of this packaging structure, as it must exhibit a wide bandwidth and low insertion loss and maximize port isolation. Consequently, a multi-branch hybrid coupler is employed in this design. As shown in Figure 5, the coupling between two adjacent GWs is achieved through five slots present in the middle metal plate. By adjusting the metal height between these coupling slots, different impedances can be obtained, and additional tunable variables can be introduced to enhance matching performance. In this design, the spacing distance between adjacent coupling slots corresponds to approximately one-quarter of the wavelength.

Based on the theoretical analysis of odd- and even-mode methods [22], the ABCD matrix can be expressed as the multiplication of matrices of multiple networks:

(1) $$\begin{array}{ll} {\left[\begin{array}{cc} A & B\\ C & D \end{array}\right]}_{e} & =\left[\begin{array}{cc} 1 & jw_{1}\tan\theta_{h}/2\\ 0 & 1 \end{array}\right]\times \left[\begin{array}{cc} 0 & \tan\theta_{s_{2}}\\ \tan\theta_{s_{2}} & 0 \end{array}\right]\times \left[\begin{array}{cc} 1 & jw_{2}\tan\theta_{h_{1}}/2\\ 0 & 1 \end{array}\right]\\ & \times \left[\begin{array}{cc} 0 & \tan\theta_{s_{1}}\\ \tan\theta_{s_{1}} & 0 \end{array}\right]\times \left[\begin{array}{cc} 1 & jw_{3}\tan\theta_{h_{2}}/2\\ 0 & 1 \end{array}\right]\times \left[\begin{array}{cc} 0 & \tan\theta_{s_{1}}\\ \tan\theta_{s_{1}} & 0 \end{array}\right]\\ & \times \left[\begin{array}{cc} 1 & jw_{2}\tan\theta_{h_{1}}/2\\ 0 & 1 \end{array}\right]\times \left[\begin{array}{cc} 0 & \tan\theta_{s_{2}}\\ \tan\theta_{s_{2}} & 0 \end{array}\right]\times \left[\begin{array}{cc} 1 & jw_{1}\tan\theta_{h}/2\\ 0 & 1 \end{array}\right] \end{array}$$

(2) $${\left[\begin{array}{cc} A & B\\ C & D \end{array}\right]}_{o}=\left[\begin{array}{cc} A_{e}(-1/P) & B_{e}(-1/P)\\ C_{e}(-1/P) & D_{e}(-1/P) \end{array}\right]$$

where *θ* is the electrical length in the waveguide, *θ_l_* = π*l* [(2/*λ*_0_)^2^ − (1/a)^2^]^1/2^, *a* is the GW width, *λ*_0_ is the free-space wavelength, and *P* = tan *θ*/2.

Additionally, the scattering matrix can be expressed as

(3) $$\begin{array}{l} S_{11}=\frac{1}{2}\Gamma_{e}+\frac{1}{2}\Gamma_{o}\;\;\;S_{21}=\frac{1}{2}T_{e}+\frac{1}{2}T_{o}\\ S_{31}=\frac{1}{2}T_{e}-\frac{1}{2}T_{o}\;\;\;S_{41}=\frac{1}{2}\Gamma_{e}-\frac{1}{2}\Gamma_{o} \end{array}$$

where Γ_e_/Γ_o_ and *T*_e_/*T*_o_ are the reflection coefficient and transmission coefficient under even/odd mode excitation, respectively.

**Figure 6:**
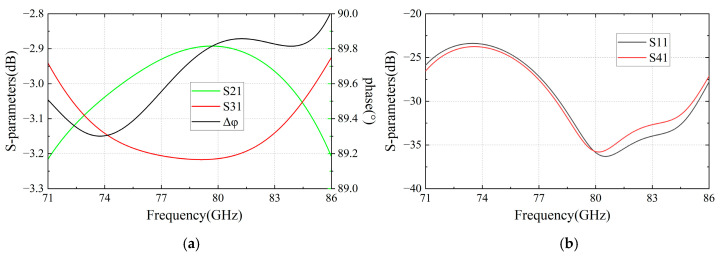
Simulated results of the designed coupler: (**a**) power distribution unbalance and output-port phase difference; (**b**) return loss and isolation levels.

Initially, using the aforementioned formulas, the preliminary dimensions can be obtained. After simulation and optimization, the final dimensions and simulated results can be obtained. As shown in Figure 6, this designed coupler operates within a frequency range of 71–86 GHz while exhibiting return loss and isolation levels exceeding 23 dB, power distribution unbalance below 0.35 dB, and an output-port phase difference of 89.6 ± 0.4°.

**Figure 7:**
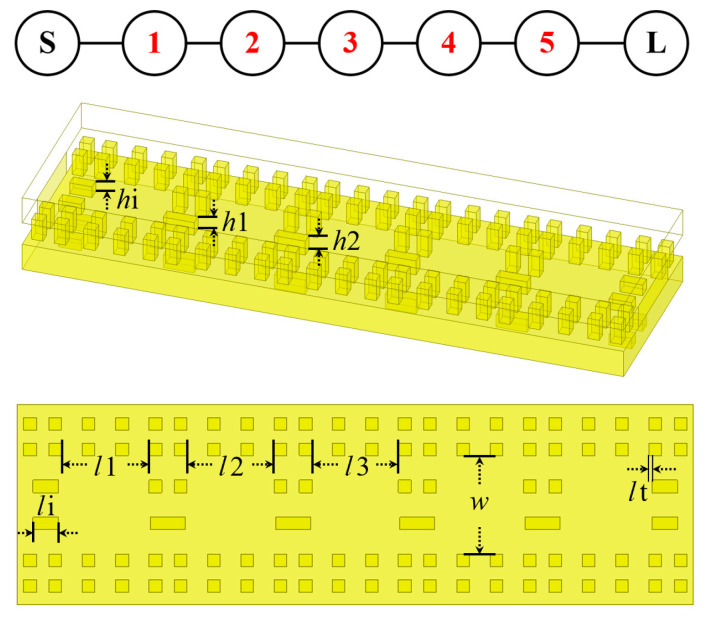
Structure and dimensions of the BPF (number 1–5 represent five resonators).

The performance of the filter determines the final frequency-selection capability of the module. Therefore, it is particularly important to design filters with low insertion loss, low-ripple, and high out-of-band rejection. In comparison to Butterworth and quasi-elliptic topologies, the Chebyshev filter balances the above indicators and is better suited for this module’s design. Consequently, this design employs a fifth-order Chebyshev topology, as depicted in Figure 7, in which an electromagnetic coupling structure connects five resonators.

**Figure 8:**
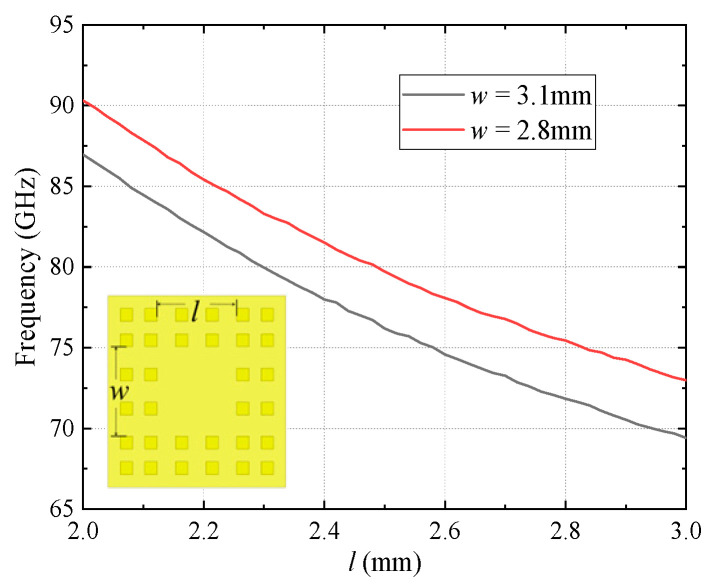
Structure of the resonator and the resonant frequency versus the resonator length.

The resonant frequency of the TE_101_ mode in the GW resonator is presented in Figure 8, where *w* and *l* are the width and length of the resonator, respectively. It can be seen that when *w* is a constant and *l* increases, the resonant frequency of the resonator decreases accordingly. In addition, due to the fact that the sidewalls of the resonant cavity created by the EBG can be equivalent to ideal electrical walls, it has properties similar to those of rectangular waveguide resonators. Therefore, the initial value of its resonant frequency can also be estimated according to Formula (4), and then the resonant frequency can be accurately designed by adjusting the dimensions.

(4) $$f_{101}=\frac{c}{2}\sqrt{{\left(\frac{1}{w}\right)}^{2}+{\left(\frac{1}{l}\right)}^{2}}$$

**Figure 9:**
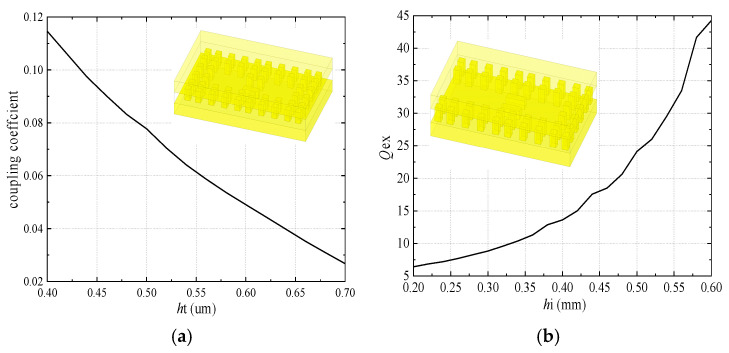
The effect of ridge height on the coupling coefficient and external quality factor: (**a**) effect of *h*t on the coupling coefficient; (**b**) effect of *h*i on the external quality factor.

Additionally, based on the model, and with copper used as the metal boundary for analysis, the simulated unloaded quality factor of the resonator is around 1800. When considering a passband range of 71–76 GHz/81–86 GHz with a return loss of −18 dB, the coupling coefficients are as follows: *m*s_1_ = 0.066/0.058, *m*_12_ = *m*_45_ = 0.0562/0.05, *m*_23_ = *m*_34_ = 0.0421/0.037, and *Q*_ex_ = 15.63/17.76 (where *m*_ij_ denotes the coupling coefficients between the *i*th and *j*th resonators) [23]. The external quality factor (*Q*_ex)_ can be adjusted by varying the heights of the ridges used for achieving electromagnetic coupling between adjacent resonators (as shown in Figure 9), and the coupling coefficient can be adjusted by changing the heights of the ridges. In addition, as the heights of the input/output-coupled ridges increase, the value of *Q*_ex_ also increases.

**Figure 10:**
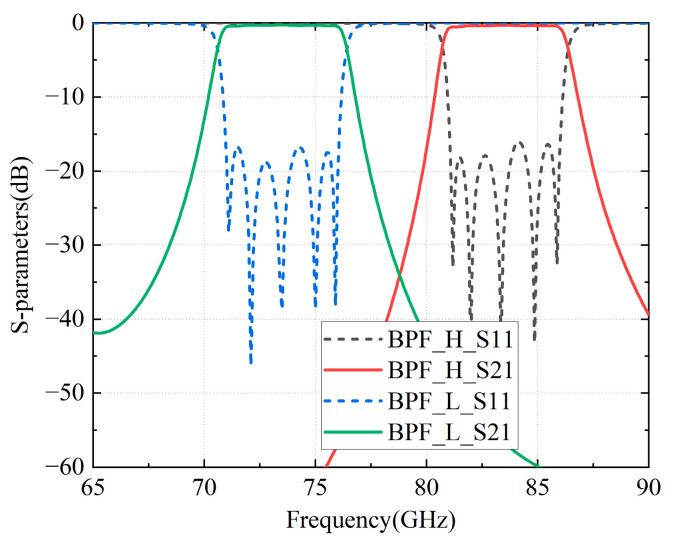
Simulated results for the BPFs.

After optimizing the coupling structures, the final dimensions and simulated results are shown in Table 1 and Figure 10, in which both bandpass filters exhibit insertion losses below 0.4 dB and return losses exceeding −18 dB while demonstrating excellent out-of-band rejection.

### 2.3. MS-GW Transition and Connecting Structure Design

**Figure 11:**
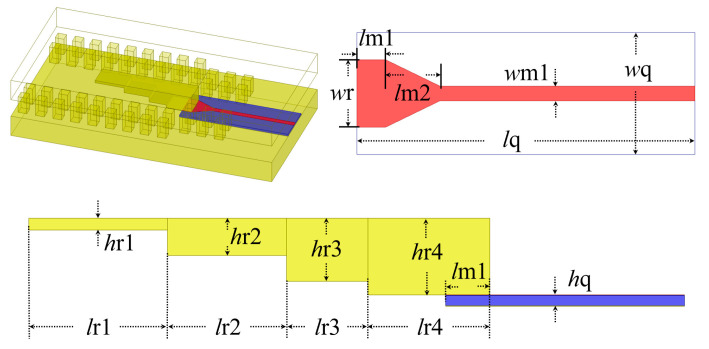
Structure and dimensions of the MS–GW transition.

The proposed transition from a MS to a GW in the amplifier application is shown in Figure 11. Within the GW, the signal undergoes conversion through multiple metal ridges of varying heights and is then tightly coupled to the MS line using a rectangular patch structure with dimensions matching those of *w*r and *l*m1 at the end of each ridge, facilitating energy transfer to the MS line. Then, the impedance is gradually transferred to a 50 Ω MS line with a width of *w*1 through a trapezoidal structure of length *l*m1. The dielectric plate material of the MS line is quartz with a thickness of 0.1 mm (*h*q = 0.1 mm). Furthermore, periodic metal square columns are introduced at the junction between each ridge end and the MS line, spaced apart by *w*q, serving as suppressors for waveguide modes.

The MS line matching is designed based on the theory of the gradient line matching theory, while the ridge waveguide matching process is designed using a fourth-order Chebyshev converter. The impedance of each order can be calculated according to the impedance converter theory. Typically, the length of the ridge waveguide chosen in the Chebyshev converter is one-quarter of the wavelength.

Based on the Chebyshev matching theory, the maximum reflection coefficient of the matching unit can be calculated as

(5) $$A=\ln(\frac{Z_{L}}{Z_{GGW}})\cdot \frac{1}{2T_{N}(\sec\theta_{M})}$$

where *Z_L_* is the load impedance (standard waveguide), and *Z_GGW_* is the input impedance (gap waveguide).

And the *θ*_M_ can be calculated by

(6) $$\theta_{M}=\frac{\pi }{2}(1-\frac{\Delta f}{2f_{0}})\;$$

where Δ*f* and *f*_0_ are the bandwidth and center frequency of the transition structure, respectively.

The impedance and the impedance of the gap waveguide sections can be computed by [24]

(7) $$Z_{\mathrm{gw}}=2\frac{\eta_{0}}{\sqrt{\varepsilon_{r}}}\frac{b}{w}$$

where *η*_0_ is the vacuum impedance, *ε_r_* is the dielectric constant of the GW filled medium, and *b* and *w* are the height and width of the GW.

According to the formulas presented above, the initial values of the transition dimensions can be determined, and the final dimensions can subsequently be obtained through optimization.

**Figure 12:**
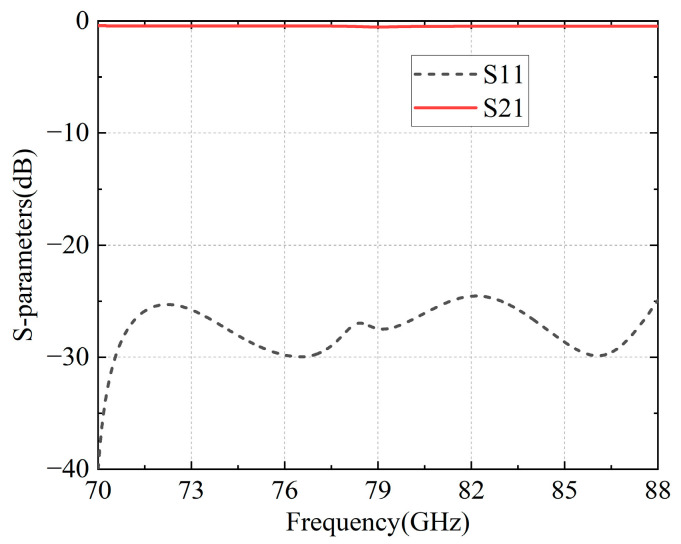
Simulated results of the MS-GW back-to-back transition.

Due to the implementation of this structure in the amplifier packaging, a back-to-back structure design is necessary. The simulated results of the final configuration are depicted in Table 2 and Figure 12, within the frequency band range of 70–88 GHz; the return loss surpasses 25 dB, while the insertion loss remains below 0.5 dB, demonstrating exceptionally low transition loss.

**Figure 13:**
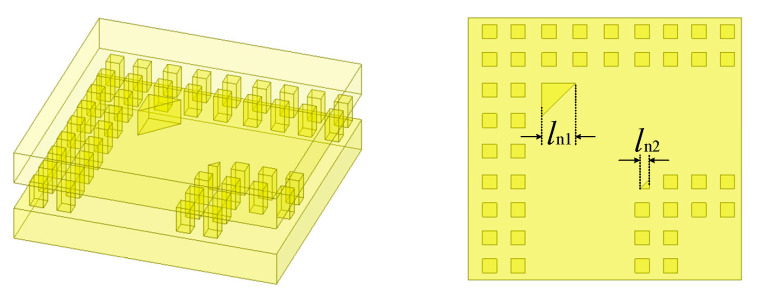
Structure and dimensions of the connecting structure. (*l*_n1_ = 0.94 mm, *l*_n2_ = 0.26 mm).

In addition, it is crucial to employ a 90° angle structure for connection in order to complete the final packaging structure design and minimize the overall volume. As illustrated in Figure 13, in this particular design, two metal nails with chamfers of side lengths *l*n1 and *l*n2 are utilized. The structure can be regarded as equivalent to a 90° bend waveguide facilitating the lossless transmission of electromagnetic energy.

**Figure 14:**
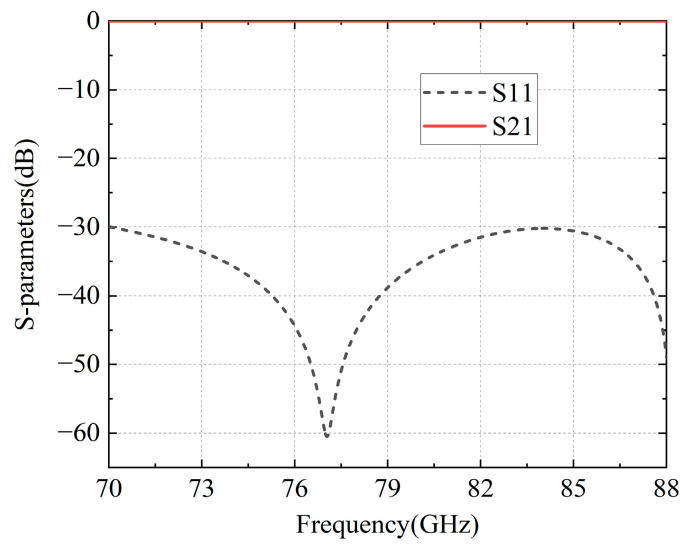
Simulated results for the connecting structure.

The simulated results are presented in Figure 14; the return loss is less than 30 dB, demonstrating excellent matching performance without causing any deterioration to the overall performance of the packaging structure.

## 3. The Fabrication and Measurement of the Packaging Structure

Due to the excellent isolation characteristics of the hybrid coupler and the absence of interference between its constituent units, it can obtain good simulated results without the necessity of intricate optimization.

**Figure 15:**
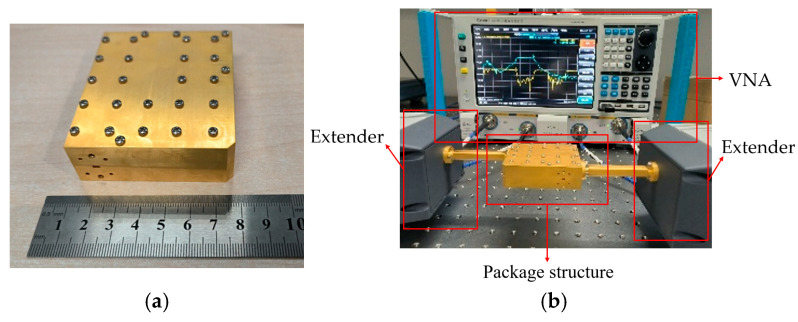
(**a**) The fabricated packaging structure; (**b**) actual measurement environment.

The final structure is depicted in Figure 3 and Figure 15a. It has three metal layers, with the MS-GW transition ridges positioned on the top and bottom metal plates, and the quartz plates and periodic EBG structures are also on both sides of the middle metal plate. Moreover, the GW ports are gradually matched to the WR10 standard waveguide through waveguide tapers.

Subsequently, the cavity of the model is fabricated using computer numerical control (CNC) technology, with copper having been chosen as the metal material to ensure the mechanical strength of the encapsulated structure. Additionally, the transition structure, which is based on a quartz dielectric substrate, is processed using a laser etching technique. Then, the quartz dielectric plate is bonded to the metal cavity using conductive adhesive, completing the assembly of the packaging structure. The process, from design to fabrication, is shown in Table 3.

**Figure 16:**
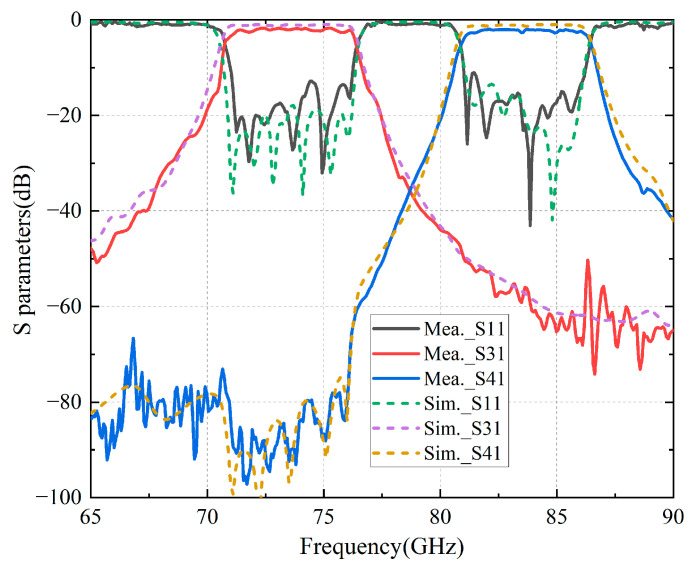
Measured and simulated results for the final packaging structure.

The fabricated model was tested using a vector network analyzer (VNA) and extenders, as shown in Figure 15b. A VNA expands its frequency range through extenders, allowing it to measure the electrical characteristics of components at frequencies between 60 and 90 GHz. The extenders are then connected to the fabricated package structure by using a straight waveguide in order to measure its S-parameters. The simulated and measured results are shown in Figure 16, and are in good agreement with the results of the simulation. Specifically, the passbands span from 70.95 GHz to 76.2 GHz and from 80.92 GHz to 86.25 GHz, respectively, featuring maximum insertion losses of 2.26 dB and 2.42 dB within these passbands, thereby demonstrating commendable electrical performance.

## 4. Conclusions

In this paper, we have proposed an E-band RF front-end packaging structure based on back-to-back GWs. By utilizing hybrid couplers as the core components and incorporating two types of fifth-order Chebyshev filters along with H-plane transitions, we have successfully achieved a complex packaging structure that is easily designable and optimizable. Meanwhile, the adoption of back-to-back structures effectively reduces the overall packaging volume. The results, subsequent to fabrication and measurement, demonstrate a passband range of 70.95–76.2 GHz and 80.92–86.25 GHz, with insertion losses below 2.26 dB and 2.42 dB, respectively, showcasing excellent electrical performance.

## Figures and Tables

**Table 1:** Dimensions of the BPFs (Unit: mm).

Parameters		*w*	*l*1	*l*2	*l*3	*l*t	*l*i	*h*i	*h*1	*h*2
value	BPF_H	2.8	2.225	2.235	2.226	0.1	0.8	0.45	0.563	0.646
	BPF_L	3.1	2.759	2.746	2.724	0.1	0.8	0.42	0.502	0.549

**Table 2:** Dimensions of the transition. (Unit: mm).

**Parameters**	***l*m1**	***l*m2**	***w*m1**	***w*r**	***w*q**	***l*q**	***h*r1**
Value	0.385	0.825	0.22	1	1.8	10	0.113
**Parameters**	***h*r2**	***h*r3**	***h*r4**	***l*r1**	***l*r2**	***l*r3**	***l*r4**
Value	0.359	0.607	0.742	1.374	1.141	0.843	1.175

**Table 3:** The design and measurement steps for the proposed packaging structure.

Stage I	Stage II	Stage III	Stage IV	Stage V
Determine the overall performance indicators, such as passband, insertion loss, etc.	1. Select the material of the substrate and the form of the transmission line 2. Design the hybrid coupler, filter and transition structure respectively	Design the signal ports and connect each component unit to simulate and optimize the model	1. Metal structures are produced using CNC technology. 2. Fabricate dielectric substrates using the photolithography process.	1. Assemble using conductive glue and screws 2. Measure the packaging structure

## Data Availability

The original contributions presented in this study are included in the article. Further inquiries can be directed to the corresponding author.
